# Supplementary material for: Upregulation of Mineralocorticoid Receptor Contributes to Development of Salt-Sensitive Hypertension after Ischemia–Reperfusion Injury in Rats
Source: Int J Mol Sci. 2022 Jul 15;23(14):7831. doi: 10.3390/ijms23147831 (PMC9324399; doi:10.3390/ijms23147831)
Supplement: Supplementary file 1 [file ijms-23-07831-s001.zip › ijms-1786536-supplementary.pdf]

Supplementary materials

Upregulation of mineralocorticoid receptor contributes to development of salt-sensitive hypertension after ischemic reperfusion injury in rats

Takumi Matsumoto, Shigehiro Doi, Ayumu Nakashima, Takeshi Ike, Kensuke Sasaki and Takao Masaki

The file includes Supplementary Figures S1, S2, S3, S4, S5.

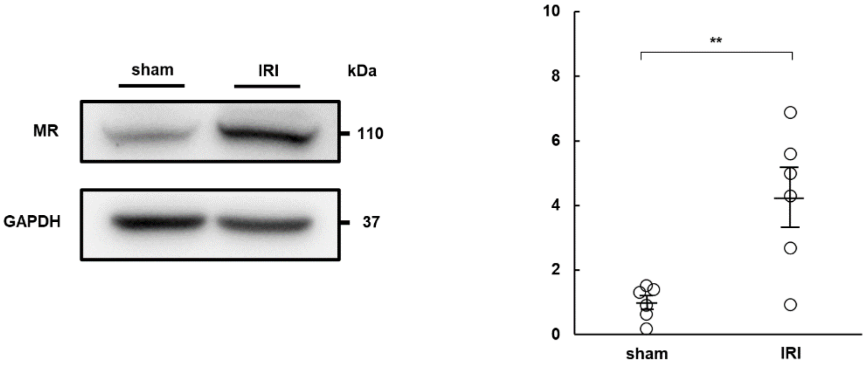

Figure S1. Expression of mineralocorticoid receptor (MR) increases in rats 6 weeks after ischemic reperfusion injury (IRI). Sprague Dawley rats underwent sham or IRI surgeries 14 days after right nephrectomy. IRI was induced by clamping the left renal artery. The rats were euthanized 6 weeks after the operations and kidney tissues were collected. Western blotting showing expression levels of MR and glyceraldehyde 3-phosphate dehydrogenase (GAPDH) in rat kidneys from the sham-and IRI-surgery groups. Relative MR protein levels in the sham and IRI groups. Band intensities were normalized to GAPDH. Values presented as mean ± standard error (n = 6 rats per group). Data analyzed by Student's *t*-test for comparisons between two groups. ** $P < 0.01$.

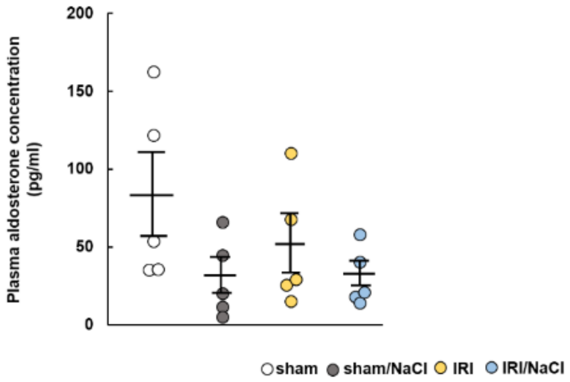

Figure S2. Increase in mineralocorticoid receptor is independent of aldosterone in ischemic reperfusion injury (IRI) rats with salt overload. Sprague Dawley rats underwent sham or IRI operations and were provided with regular drinking water or 1.0% NaCl solution for 6 weeks. Graph shows plasma aldosterone concentration. Values presented as mean ± standard error (n = 5 rats per group). Data analyzed by one-way analysis of variance followed by Tukey's test.

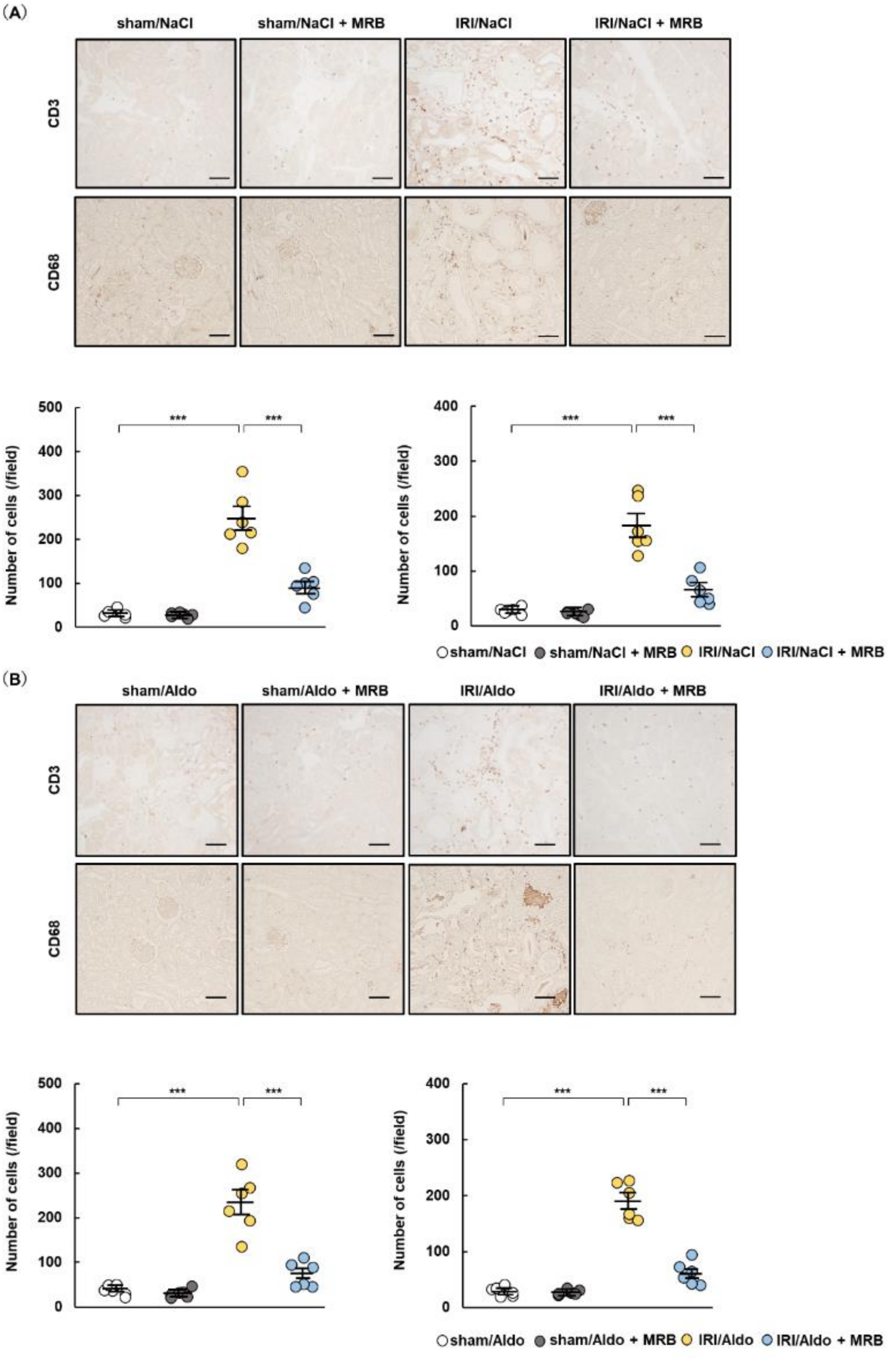

(A)
sham/NaCl
sham/NaCl + MRB
IRI/NaCl
IRI/NaCl + MRB
CD3
CD68
Number of cells (/field)
500
400
300
200
100
0
***
***
Number of cells (/field)
400
300
200
100
0
***
***
sham/NaCl
sham/NaCl + MRB
IRI/NaCl
IRI/NaCl + MRB
(B)
sham/Aldo
sham/Aldo + MRB
IRI/Aldo
IRI/Aldo + MRB
CD3
CD68
Number of cells (/field)
500
400
300
200
100
0
***
***
Number of cells (/field)
400
300
200
100
0
***
***
sham/Aldo
sham/Aldo + MRB
IRI/Aldo
IRI/Aldo + MRB

(C)

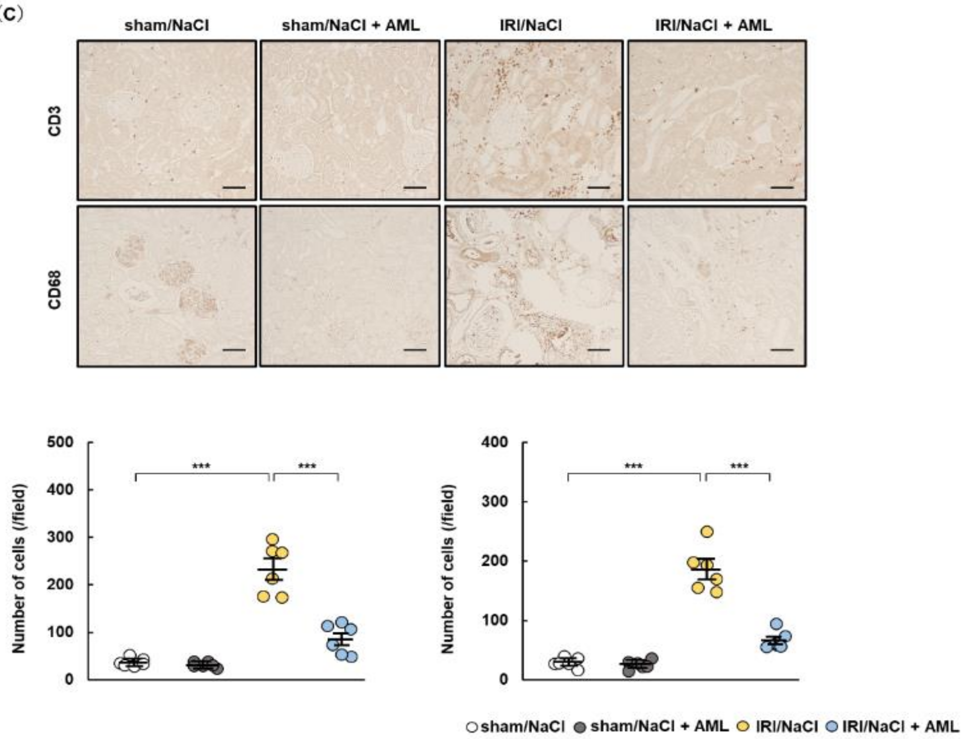

(D)

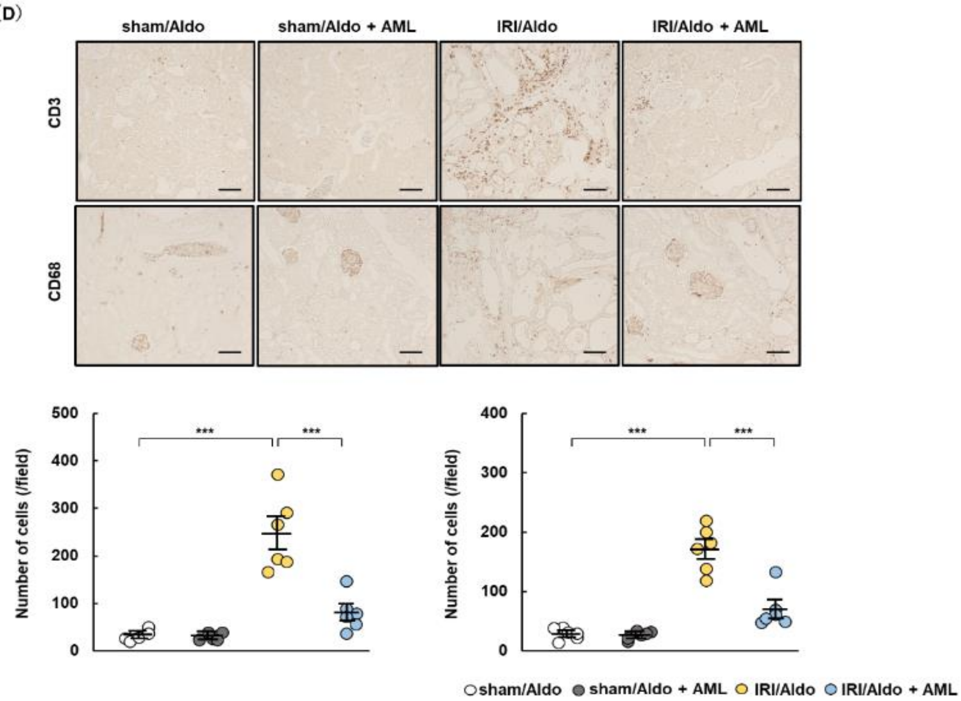

Figure S3. Esaxerenone and amiloride ameliorates (AML) inflammation in ischemic reperfusion injury (IRI) rats with salt overload or aldosterone infusion. Sprague Dawley

rats underwent sham or IRI operations and were provided with 1.0% NaCl solution or infused with aldosterone. The non-steroidal mineralocorticoid receptor blocker esaxerenone, AML, or vehicle was administered to sham and IRI rats for 6 weeks. Representative images of immunohistochemical staining for CD3 and CD68 show inflammatory changes in renal tissue (scale bar = 100 μm). Esaxerenone treatment in sham and IRI rats provided with (A) 1.0% NaCl solution and (B) infused with aldosterone. AML treatment in sham and IRI rats provided with (C) 1.0% NaCl solution and (D) infused with aldosterone. The numbers of CD3- or CD68-positive cells were quantified for each group from stained images and presented in the graphs. Data presented as mean ± standard error (n = 6 rats per group). Data analyzed by one-way analysis of variance followed by Tukey's test. *** $P < 0.001$.

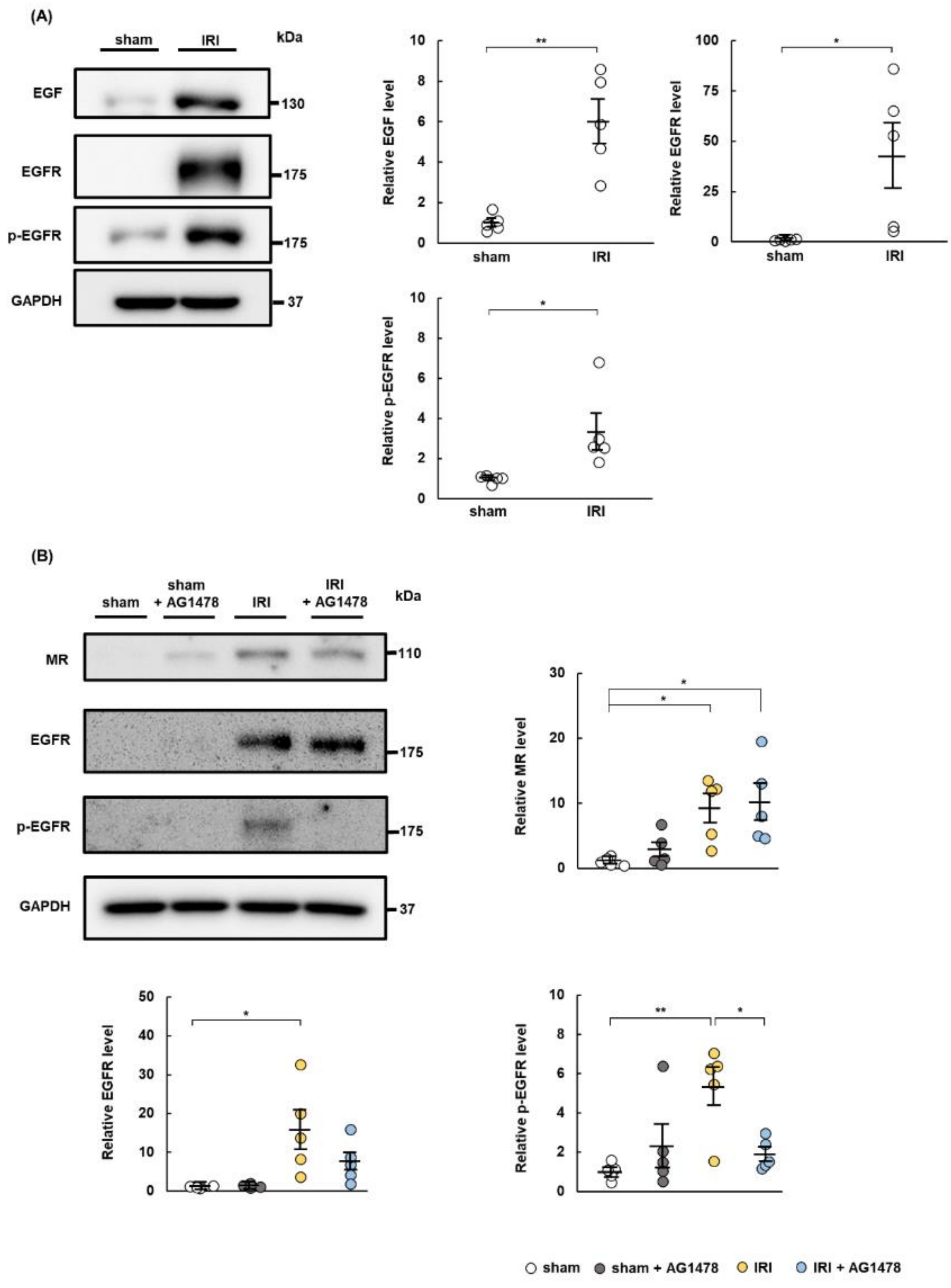

Figure S4. AG1478 administration do not affect expression level of mineralocorticoid receptor (MR) in ischemic reperfusion injury (IRI) rats. To determine if epithelial growth factor (EGF) was involved in the increased expression of MR in IRI rats, rats were

allocated into four groups 7 days after sham or IRI operations (n = 5 per group each): Group 1, intraperitoneal injection of vehicle after sham operation (sham rats); Group 2, intraperitoneal injection of the EGF receptor (EGFR) tyrosine kinase inhibitor AG1478 (10 mg/kg/day, MedChemExpress Monmouth Junction, NJ, USA) in sham rats (sham + AG1478 rats); Group 3, vehicle administration in IRI rats (IRI rats); and Group 4, AG1478 administration in IRI rats (IRI + AG1478 rats). Vehicle and AG1478 were administered daily and rats were euthanized by cardiac puncture under deep anesthesia at day 7 after sham or IRI operation. Dimethyl sulfoxide, polyethylene glycol 300, polyoxyethylene sorbitan monooleate, and saline were used as the AG1478 vehicle. The following primary antibodies were used: anti-EGF antibody (1:2,500; AB_2095977; NBP1-19806, Novus Biologicals, Littleton, CO, USA); anti-EGFR antibody (1:2,500; AB_869579; ab52894; Abcam Cambridge, UK); and anti-phospho (p)-EGFR (1:2,500; AB_331701; 2234S; Cell Signaling Technology, Danvers, MA, USA). (A) Representative western blots showing expression levels of EGF, EGFR, p-EGFR, and glyceraldehyde 3-phosphate dehydrogenase (GAPDH) in renal tissue of rats 7 days after sham or IRI operation. (B) Representative western blots showing expression levels of EGFR, p-EGFR, MR, and GAPDH. GAPDH was used as a loading control. Graphs show relative expression of each protein. Data presented as mean ± standard error (n = 5 rats per group). Comparisons between two groups were tested using Student's *t*-test and multiple comparisons were tested by one-way analysis of variance followed by Tukey's test. * $P < 0.05$, ** $P < 0.01$.

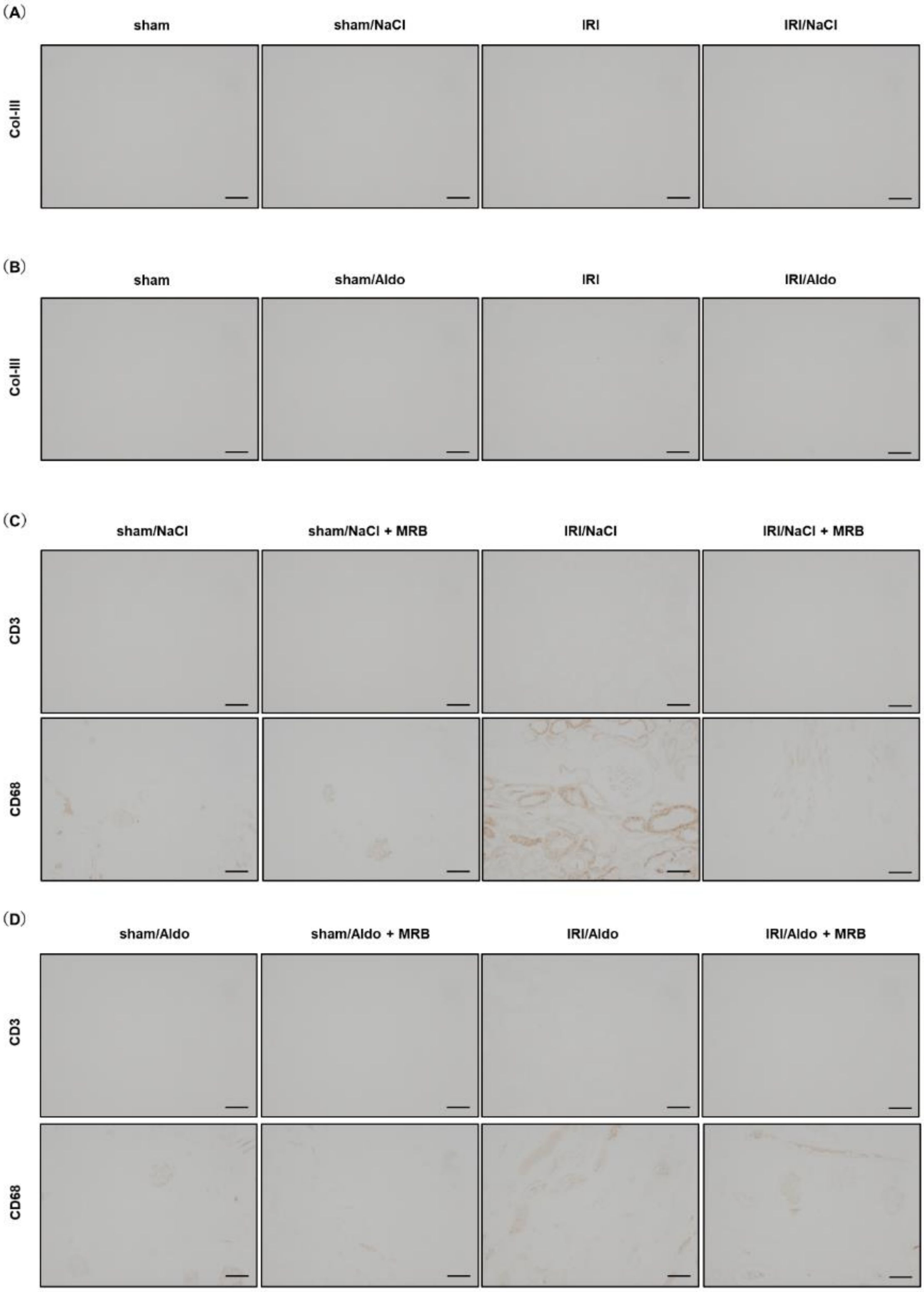
(A)
sham
sham/NaCl
IRI
IRI/NaCl
Col-III
(B)
sham
sham/Aldo
IRI
IRI/Aldo
Col-III
(C)
sham/NaCl
sham/NaCl + MRB
IRI/NaCl
IRI/NaCl + MRB
CD3
CD68
(D)
sham/Aldo
sham/Aldo + MRB
IRI/Aldo
IRI/Aldo + MRB
CD3
CD68

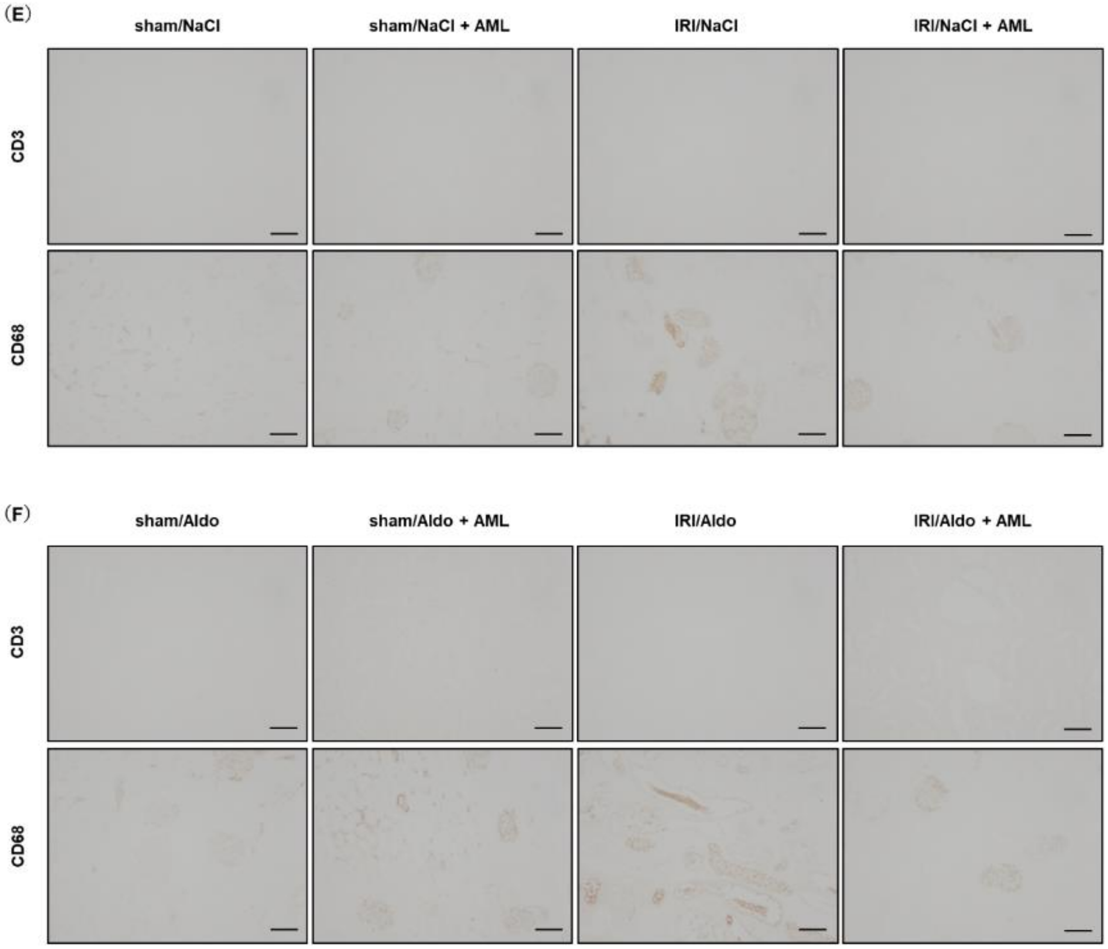

Figure S5. Immunohistochemistry images for controls without primary antibody. Immunohistochemistry images for non-specific antibody. Immunohistochemical staining of renal tissue without primary collagen type 3 (Col-III) antibody presented in (A) Figure 2C and (B) Figure 3C. Immunohistochemical staining of renal tissue without primary CD3 and CD68 antibodies presented in (C) Figure S3A, (D) Figure S3B, (E) Figure S3C, and (F) Figure S3D. Scale bars = 100 µm.
